# Complete Genome Sequence of *Bacillus thuringiensis* Serovar Tolworthi Strain Pasteur Institute Standard

**DOI:** 10.1128/genomeA.00710-15

**Published:** 2015-07-02

**Authors:** Kohzo Kanda, Kaede Nakashima, Yukio Nagano

**Affiliations:** a Department of Applied Biochemistry and Food Science, Faculty of Agriculture, Saga University, Saga, Japan; b Analytical Research Center for Experimental Sciences, Saga University, Saga, Japan

## Abstract

The genome sequence of *Bacillus thuringiensis* serovar tolworthi strain Pasteur Institute Standard was determined. The genome consists of a 5.9-Mb chromosome and eight plasmids, one of which is linear. The second largest plasmid (293 kb) carries the genes encoding insecticidal proteins.

## GENOME ANNOUNCEMENT

*Bacillus thuringiensis* is a Gram-positive bacterium that produces insecticidal δ-exotoxins called crystal (Cry) proteins. *B. thuringiensis* serovar tolworthi is a representative serovar that has been studied for more than 50 years. However, only the draft genome sequence of serovar tolworthi strain Na205-3 is available (1). Here, we report a complete genome sequence of *B. thuringiensis* serovar tolworthi strain Pasteur Institute Standard, which was introduced at Saga University more than 20 years ago.

Analysis of purified DNA with the PacBio RS II system yielded 103,505 subreads with a mean length of 5.5 kb (covering the genome 70-fold). The reads were corrected with Sprai version 0.9.9.2 (http://zombie.cb.k.u-tokyo.ac.jp/sprai/) and assembled with Celera Assembler version 8.3rc1 (2). Because the coverage depth of PacBio reads was low (~25-fold) in the region near the possible terminal of chromosomal replication, it was difficult to assemble this region by PacBio reads only. Therefore, we constructed hybrid assemblies using both PacBio and Illumina reads (>7 billion nucleotides covering the genome >1,000-fold) with SPAdes version 3.5.0 (3). The contigs obtained from both methods were assembled manually, and the assembled sequences were polished with Quiver (https://github.com/PacificBiosciences/GenomicConsensus/blob/master/doc/HowToQuiver.rst) using the PacBio reads. Because most sequence errors in PacBio reads are insertions/deletions (indels) (4) and mapping of Illumina reads to the assembled sequence indicated the presence of indel changes, particularly in the region near the terminal of chromosomal replication, we corrected these changes based on the mapping results.

The genome consisted of a 5,896,839-bp circular chromosome, a 14,827-bp linear plasmid pKK6, and seven circular plasmids: pKK1 (437,451 bp), pKK2 (293,217 bp), pKK3 (130,548 bp), pKK4 (54,355 bp), pKK5 (23,773 bp), pKK7 (11,769 bp), and pKK8 (7812 bp). The MiGAP program (http://www.migap.org/index.php/en) predicted 7,044 coding sequences, 112 tRNAs, and 36 rRNAs. The conserved syntenies over the entire chromosome were present in several strains of *B. thuringiensis* (e.g., serovar chinensis strain CT-43 [5]). The linear plasmid pKK6 was shown to contain inverted terminal repeats at both extremities and had phage-like characteristics, as predicted by the phage search tool PHAST (6). Because the coverage depth of Illumina reads at both extremities was low (~10-fold), we confirmed these sequences using the Sanger method.

The plasmid pKK2 was shown to contain genes similar to insecticidal proteins, VIP2, Cry2Aa, Cry1Ia, and Cry1Ea, although the Cry1Ia protein may be truncated. Plasmids similar to pKK2 are present in several strains of *B. thuringiensis*: pBMB299 in serovar kurstaki strain HD-1 (http://bacteria.ensembl.org/bacillus_thuringiensis_serovar_kurstaki_str_hd_1_gca_000717535_1/Info/Index/; 82% query coverage), pBMB293 in serovar kurstaki strain YBT-1520 (http://bacteria.ensembl.org/bacillus_thuringiensis_serovar_kurstaki_str_ybt_1520_gca_000688795_1/Info/Index/; 82% query coverage), pIS56-285 in subspecies thuringiensis strain IS5056 (81% query coverage) (7), pCT281 in serovar chinensis strain CT-43 (81% query coverage) (5), and pBMB267 in serovar galleriae strain HD-29 (66% query coverage) (8). A BLAST search suggested that *B. thuringiensis* serovar tolworthi strain Na205-3 (1) contained sequences that were partially similar to the plasmid pKK2.

### Nucleotide sequence accession numbers.

The complete genome sequence of *B. thuringiensis* serovar tolworthi strain Pasteur Institute Standard has been deposited in DDBJ/EMBL/GenBank under the accession numbers AP014864 (chromosome) and AP014865 to AP014872 (plasmids).
